# Shared Decision‐Making and Body Mass Index in Australian Antenatal Care: An Exploratory OPTION12 Evaluation

**DOI:** 10.1111/hex.70107

**Published:** 2024-11-17

**Authors:** Madeline Hawke, Linda Sweet, Julie Considine

**Affiliations:** ^1^ School of Nursing and Midwifery Deakin University Geelong Victoria Australia; ^2^ Centre for Quality and Patient Safety Research – Western Health Partnership Sunshine Victoria Australia; ^3^ Centre for Quality and Patient Safety Research – Eastern Health Partnership Box Hill Victoria Australia

**Keywords:** decision making, shared, midwifery, midwives, obesity, maternal, obstetrician, weight prejudice

## Abstract

**Background:**

Shared decision‐making is recommended as a person‐centred approach to decision‐making in antenatal care. Little is known about the implementation of shared decision‐making in antenatal care.

**Objective:**

An exploratory study to understand how shared decision‐making is implemented in antenatal clinics and whether body mass index influences maternity clinicians’ use of shared decision‐making when providing antenatal care for women.

**Methods:**

Twenty‐six antenatal clinic consultations were audio‐recorded with maternity clinicians and women with body mass index ≥ 35 kg/m^2^, and a comparison group of women with body mass index 18.5–24.9 kg/m^2^. Data were analysed quantitatively using the OPTION12 scale. Narrative case studies are presented to compare shared decision‐making behaviour related to induction of labour.

**Results:**

Twelve clinicians and 26 pregnant women were recruited to the study. The total scores ranged from 0 to 24, with a mean score of 9 and a median of 9.5 indicating low implementation of shared decision‐making by clinicians and limited involvement of women in decision‐making. No difference was observed in OPTION12 scores in decision‐making for women by body mass index.

**Conclusion:**

This study suggests that shared decision‐making is limited in the antenatal clinic setting for all women, regardless of body mass index. Further research is required to confirm the findings of this exploratory study.

**Patient or Public Contribution:**

The perspectives of women with body mass index ≥ 35 kg/m^2^ informed many aspects of this study including the language/terminology adopted by researchers. A consumer group reviewed the language used in the study materials, to ensure readability and avoidance of stigmatising terminology.

## 1 Introduction

Decision making in maternity care has historically been paternalistic, where the maternity clinician maintains authority, and women's decisions are distilled into either consenting or declining recommended care [1, 2]. Shared decision making in maternity care is proposed as a person‐centred alternative to paternalistic decision making, involving a collaborative partnership between the clinician and pregnant woman [2, 3, 4]. Shared decision making in maternity care is defined as an enquiry by the pregnant woman and clinician in the form of a dialogue, to determine a pathway of care that aligns with the woman's preferences, where the pregnant woman is supported in decision making by the clinician's knowledge and expertise [2]. Shared decision making does not replace the legal doctrine of informed consent but precedes and enhances it, with a dialogue involving alternative options of care [2, 5]. Decisions most suited to shared decision making are those where clinical equipoise exists or where multiple options could be considered. In antenatal care, there are many opportunities to employ shared decision making from consideration of investigations, planning for mode and timing of birth, or where the woman's preferences or values conflict with recommended care [2]. Shared decision‐making reduces the risk of physical or psychological trauma relating to childbirth and improves the experience of care across the perinatal continuum [3, 6]. Shared decision‐making is a key component of contemporary health policy in Australia and central to the Australian Commission on Safety and Quality in Health Care (ACSQHC) National Safety and Quality Health Service Standards (NSQHSS) [7]. All public and private hospitals in Australia are accredited against the NSQHSS, meaning that implementation of shared decision‐making is mandated in Australian hospital‐based maternity care [8].

Women carrying excess weight represent over half of pregnant women in the United States, the UK and Australia, and carry a significantly higher risk of complications in pregnancy compared to those with lower body weight [9, 10, 11]. In pregnancy, excess weight is calculated using the body mass index (BMI: body weight in kilograms divided by height in metres squared). Despite criticism of the BMI as an inaccurate measure of health, it remains the primary tool for determining weight‐related risk in pregnancy [12, 13, 14]. The BMI indicators used in numerous Australian guidelines to determine a woman's risk of developing weight‐related complications in the perinatal period are shown in Table 1 [16, 17, 18].

**Table 1:** World Health Organisation's body mass index (BMI) classification.

Classification	BMI (kg/m²)	Risk of comorbidities
Underweight	< 18.5	Low (but risk of other clinical problems increases)
Normal range	18.5–24.9	Average
Pre‐obese	25.0–29.9	Increased
Obese class I	30.00–34.9	Moderate
Obese class II	35.0–39.99	Severe
Obese class III	> 40.00	Very severe

*Source:* [15].

Recommended changes to antenatal care for women with BMI ≥ 30 kg/m^2^ are variable and differ across state, national and international guidelines, and there are no standard recommendations for several aspects of care [19, 20]. Many recommendations for antenatal care of women with BMI ≥ 30 kg/m^2^ are based on limited evidence, with 12 of the 16 recommendations being consensus‐based in the Royal Australian and New Zealand College of Obstetrics and Gynaecology guidelines for Management of Obesity in Pregnancy [21]. A BMI of ≥ 35 kg/m^2^ (Class II obesity) is often the threshold where changes to recommended care are instituted to manage increased risk of conditions such as gestational diabetes, pre‐eclampsia and stillbirth [9, 20, 22]. Changes to recommended care for women with BMI ≥ 35 kg/m^2^ include increased frequency of tests for diabetes and pre‐eclampsia, increased frequency of ultrasounds in the third trimester and consideration of induction of labour before term [20, 23]. Given the limited evidence guiding recommended care for women with BMI ≥ 35 kg/m^2^, the decision to undergo additional tests and interventions provides an opportunity for the implementation of shared decision making by maternity clinicians.

Women with BMI ≥ 35 kg/m^2^ experience stigma associated with excess weight. Weight stigma involves attributing negative stereotypes to an individual based on weight and affects interactions between clinicians and the pregnant woman [24, 25, 26]. Women with BMI ≥ 35 kg/m^2^ have reported interactions with maternity clinicians during pregnancy as highly stigmatising and discriminatory [26, 27, 28, 29, 30, 31, 32]. Stigmatising experiences have been documented across multiple aspects of antenatal care, including women being told by doctors that they were putting their unborn child at risk because of their weight, being treated disrespectfully by sonographers during ultrasounds, and being refused or coerced into care [31, 32, 33, 34, 35] There is evidence that maternity clinicians' communication is less person‐centred with women with BMI ≥ 35 kg/m^2^, compared to women with BMI of 18.5–24.9 kg/m^2^ [36]. As shared decision making is a person‐centred approach to healthcare decision making, it is helpful to understand whether clinicians provide the same opportunity for shared decision making in antenatal consultations with women with BMI ≥ 35 kg/m^2^ as for women with BMI 18.5–24.9 kg/m^2^. A number of studies have been undertaken exploring shared decision‐making in health care [37, 38], but few have assessed the use of shared decision making by midwives and doctors in antenatal clinics [39, 40], and no studies have explored maternity clinicians' use of shared decision making specifically with women with BMI ≥ 35 kg/m^2^. Shared decision making has demonstrated benefits for women in pregnancy; however, maternal BMI may influence clinicians' use of shared decision‐making in antenatal care. The aim of this study was to explore the implementation of shared decision‐making in antenatal clinic, both for women with BMI ≥ 35 kg/m^2^ and women BMI 18.5–24.9 kg/m^2^, and to understand whether maternal BMI influences access to shared decision making in antenatal care.

## 2 Materials and Methods

This paper is reporting on a quantitative OPTION12 evaluation of audio‐recorded antenatal clinic appointments. This was part of a larger convergent mixed‐methods study that included audio‐recorded antenatal clinic appointments with maternity clinicians and women with BMI 18.5–24.9 kg/m^2^ and BMI ≥ 35 kg/m^2^ and follow‐ up, semi‐structured interviews with maternity clinicians and women with BMI ≥ 35 kg/m^2.^ The perspectives of women with BMI > 35 kg/m^2^ informed many aspects of this study including the language/terminology adopted by researchers. A consumer group was involved in reviewing the language used in the Participant Information and Consent Form (PICF) and study materials, to ensure readability and avoidance of stigmatising terminology.

### 2.1 Ethics

The study was conducted at two hospitals, one in the eastern suburbs and one in the western suburbs of metropolitan Melbourne, Australia. Ethics approval was received from the Royal Melbourne Hospital Human Research Ethics Committee (HREC/94723/MH‐2023) in May 2023 through the National Mutual Acceptance scheme, allowing multi‐site research to be conducted [41]. Site governance and reciprocal approval were gained through a university and the study hospitals.

Data storage and management complied with all relevant standards and codes including Deakin University Research Data and Primary Materials Management procedure and the Australian Code for the Responsible Conduct of Research [42, 43]. Complete confidentiality and anonymity were not possible in the context of this study, as the first author knew the names and faces of participants. However, data were de‐identified following transcription of the audio recordings. Personally identifying information and contextual identifiers were removed before data analysis [44].

### 2.2 Participants and Recruitment

Purposive sampling was used to recruit participants, based on their willingness to participate, study eligibility, and clinical role (clinicians), or attendance (women) at one of the two study sites for antenatal care. The researcher aimed to record appointments with one woman with BMI ≥ 35 kg/m^2^ and one woman with BMI 18.5–24.9 kg/m^2^ per clinician to compare shared decision‐making with women of different body sizes. Maternity clinicians were eligible if they were a midwife or doctor providing primary care in antenatal clinic. Women were eligible to participate if they were ≥ 18 years old, spoke and understood English, and had a BMI of either 18.5–24.9 kg/m^2^ or ≥ 35 kg/m^2^. Consideration was given to the risk of the Hawthorne effect, whereby participants change their behaviour as a result of being observed [45]. To minimise the potential Hawthorne effect, audio recording was chosen as a minimally invasive method of observation and an abridged study aim was provided in the PICF and verbal communication to participants about the study, focussing more broadly on communication with women of different body sizes rather than explicitly referencing shared decision‐making.

Maternity clinicians were provided with a PICF and verbal explanation of the study by the first author. A demographic questionnaire was completed by each maternity clinician including gender and clinical role. Following maternity clinician consent, women booked to see participating clinicians were approached in the antenatal clinic waiting room and invited to participate. Women were provided with a PICF and verbal explanation of the study by the first author. Those interested in participating were asked to complete a screening questionnaire where eligibility was determined based on reported age, booking height and weight, and current height and weight (to calculate BMI). Women's eligibility to participate was based on their BMI at the time of screening, however, height, weight and BMI at booking (the first antenatal appointment) were also collected as their booking BMI could influence shared decision‐making. Additional demographic information was collected including primary language, educational attainment, and pregnancy gestation. Participation in the study was voluntary, and written, informed consent was obtained from all participants and any partners, support people, or student clinicians present during the consultation.

### 2.3 Data Collection

Following recruitment and consent, the woman was provided with a handheld digital recorder to audio record the antenatal appointment. The researcher remained in the waiting room during the consultation. Giving women control of the handheld digital recorder was intended to provide an added layer of autonomy for women participating in the study, and women were advised they could pause or stop the recording at any time during the consultation if they did not want sensitive information recorded. However, all participants recorded the entire appointment without pause. After the clinic consultation, the recorder was returned to the researcher. As this was an exploratory study, all index problems were included as part of the research instead of a focussed study of a specific index problem (e.g., shared decision‐making about induction of labour). Further, the diversity of index problems addressed in an antenatal clinic consultation is reflective of real‐world clinical practice.

### 2.4 Data Analysis

Recordings were transcribed verbatim by the first author or a professional transcription service. The transcriptions from the professional transcription service were checked for accuracy by the first author. Audio recordings were then analysed using the OPTION12 scale [46] to measure shared decision‐making.

#### 2.4.1 OPTION12 Scale

The OPTION12 scale is a theoretically grounded observer measure of shared decision‐making in clinical practice [47]. When compared with other instruments designed to measure shared decision‐making, the OPTION12 scale has the most robust evidence for validity, determined by a 2020 systematic review of all shared decision‐making instruments [48]. The OPTION12 scale was chosen for this study for its validity and to align with the other study of shared decision‐making in antenatal care completed by Fersini et al. [39]. The OPTION12 scale scores clinical encounters against 12 items, deemed key criteria for implementation of shared decision‐making in practice (See Table 2) [49]. The decision‐making interaction that is scored, known as the index problem, is identified by choosing the decision‐making interaction where the highest degree of women's involvement is achieved by the clinician [46]. The OPTION12 scoring also takes into account duration and type of consultation, and whether a consultation involves a new problem, review of an existing problem, or a composite discussion of new problems and reviewed problems [46].

**Table 2:** OPTION 12 instrument [46].

The clinician…
1. Draws attention to an identified problem as one that requires a decision‐making process
2. The clinician states that there is more than one way to deal with the identified problem (‘equipoise’).
3. The clinician assesses the patient's preferred approach to receiving information to assist decision making
4. The clinician lists ‘options’, which can include the choice of ‘no action’.
5. The clinician explains the pros and cons of options to the patient (taking ‘no action’ is an option).
6. The clinician explores the patient's expectations (or ideas) about how the problem(s) are to be managed.
7. The clinician explores the patient's concerns (fears) about how problem(s) are to be managed.
8. The clinician checks that the patient has understood the information
9. The clinician offers the patient explicit opportunities to ask questions during the decision‐making process.
10. The clinician elicits the patient's preferred level of involvement in decision‐making.
11. The clinician indicates the need for a decision‐making (or deferring) stage.
12. The clinician indicates the need to review the decision (or deferment).
Scoring:
0 The behaviour is not observed
1 A minimal attempt is made to exhibit the behaviour
2 The behaviour is observed and a minimum skill level is achieved
3 The behaviour is observed to a good standard
4 The behaviour is observed to a very high standard

Before the commencement of scoring, the authors completed the OPTION12 training pack provided with this instrument [46]. All OPTION12 scores were calculated by the first author, with the second rating completed by either the second or third author. Data were analysed using IBM SPSS Statistics for Windows, Version 29. Interrater agreement was calculated using a weighted Cohen's Kappa coefficient, with linear weights for each item of the OPTION12 scale. The degree of shared decision‐making in antenatal consultations was calculated by adding the scores for each item for each rater, calculating the mean of the two total scores, and then transforming the mean score to a scale of 0–100, resulting in an OPTION12 score between 0 and 100 [38]. The researchers who developed the OPTION12 scale identified that scores of 0 or 1 for each item of the scale indicate a low level of patient involvement in decision‐making [46]. This has been further elaborated upon by later OPTION12 research where a total score of < 35 is considered to indicate a low level of shared decision‐making [49, 50, 51]. Inferential statistical analysis was not undertaken due to the small sample size and non‐probability sampling.

### 2.5 Case Studies

To allow for comparison of approaches to shared decision‐making, two narrative case studies are presented. Narrative case studies provide a multi‐faceted understanding of a complex issue in a real‐life context [52, 53]. Narrative case studies are commonly used in health and social science research to provide experience‐near descriptions of clinical interactions [53]. The case studies in this paper were deductively selected to demonstrate the shared decision‐making behaviour of clinicians. Following OPTION12 scoring, two narrative case studies were selected from the compiled consultation transcripts to provide examples of interactions which scored highest and lowest on the OPTION12 scale, involving the same index problem and participants with similar characteristics.

## 3 Results

### 3.1 Participant Characteristics

**Figure 1:**
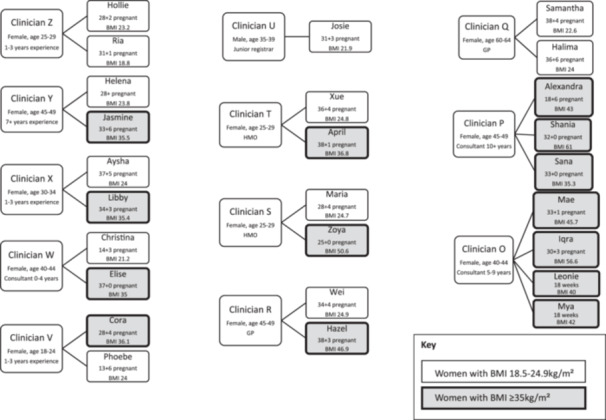
Participant interaction.

Twelve clinicians and 26 pregnant women were recruited to the study. The demographic details of all participants are presented in Table 3. Pseudonyms were assigned to participants using a random name generator for women and codenames for clinicians, to protect participant anonymity [54]. Four midwives and eight doctors agreed to participate in the study, most clinicians identified as female. The 26 pregnant women ranged in age from 22 to 39 years of age. There were 14 women with BMI ≥ 35 kg/m^2^ and 12 women with BMI 18.5–24.9 kg/m^2^. Women had varied educational backgrounds, and six participants spoke English as a second language. Both hospital sites have specialist clinics for women with high BMI. Participants were recruited for this study from both the standard clinic (*n* = 21) and specialist clinic for women with high BMI (*n* = 5). It was not possible to pair all clinicians with both women with BMI 18.5–24.9 kg/m^2^ and BMI ≥ 35 kg/m^2^, however, pairing was achieved with seven of the 12 clinicians (see Figure 1). The average length of consultations was 20 min (range 8–44 min) and the majority of appointments were composite, involving discussion of new and previously reviewed problems.

**Table 3:** Participant characteristics.

Women's demographics	Total *n* = 26
Mean age (range)	32 (22–39)
Gestation range	13 + 6 to 38 + 4
English as a second language	6
First pregnancy	11
Education:	
High school	6
Diploma	6
Undergraduate degree	9
Postgraduate degree	5
Body mass index (BMI):	
BMI ≥ 35 kg/m^2^	14
BMI 18.5–24.9 kg/m^2^	12
**Clinician demographics**	**Total n** = **12**
Female:male	11:1
Clinical role:	
Midwife	4
Obstetrician‐Gynaecologist	3
Registrar	1
House Medical Officer	2
General practitioner	2
**Clinician–Woman encounters**	**26**
Type of consultation:	
New	1
Review	4
Composite	21
Minutes of recorded data	533

### 3.2 OPTION12 Scores

None of the consultations in this study achieved a OPTION12 score ≥ 35, indicating that shared decision‐making behaviour was low in all recorded consultations (Table 4). Most interactions scored 0 or 1 per item, indicating that shared decision making was not observed, or a minimal attempt was made to exhibit shared decision‐making behaviour [46]. In 12 of the interactions, clinicians scored 2 across some items, indicating a minimum skill level of shared decision making was achieved [46]. None of the clinicians exhibited the shared decision‐making skill to a good (score of 3) or very high (score of 4) standard as defined by Elwyn et al. in the OPTION12 manual [46]. The total OPTION12 scores in this study ranged from 0 to 24, with a mean score of 9 and a median of 9.5 [50]. Inter‐rater agreement was calculated with a mean‐weighted Cohen's kappa of 0.761, demonstrating substantial agreement between raters [55].

**Table 4:** OPTION12 scoring.

Encounter	Woman's BMI (kg/m^2^)	Index problem	Composite, review or new appointment	Consultation duration (mins)	1	2	3	4	5	6	7	8	9	10	11	12	Rater 1 and Rater 2 score	Mean score	OPTION 12 score*
Helena & Clinician Y	23.8	Iron studies pathology test	Composite	15.13	1	1	0	0	0	0	0	0	0	0	0	0	2	2	**4**
					1	0	0	0	1	0	0	0	0	0	0	0	2		
Jasmine & Clinician Y	35.5	GBS swab	Composite	20.07	2	1	0	2	0	0	0	0	0	0	1	0	6	6.5	**14**
					2	1	0	1	1	0	0	0	0	0	2	0	7		
Cora & Clinician V	36.1	Whooping cough vaccination	Composite	18.13	2	1	0	0	0	0	0	0	0	0	0	0	3	3.5	**7**
					2	1	0	0	1	0	0	0	0	0	0	0	4		
Phoebe & Clinician V	24	Cervical screening test	Composite	36.46	2	2	0	1	1	0	0	0	0	0	0	0	6	6.5	**14**
					2	2	0	1	2	0	0	0	0	0	0	0	7		
Mae & Clinician X	45.6	Induction of labour	Composite	16.08	1	0	0	0	0	0	0	0	0	0	0	0	1	1	**2**
					1	0	0	0	0	0	0	0	0	0	0	0	1		
Christina & Clinician W	21.2	Timing of birth	Composite	23.39	2	1	0	1	0	1	1	0	1	0	1	0	8	7	**15**
					2	1	0	1	1	0	0	0	0	0	1	0	6		
Elise & Clinician W	35	Induction of labour	Composite	31.34	2	2	0	2	1	0	1	0	0	0	2	2	12	11.5	**24**
					2	2	0	2	1	0	0	0	0	0	2	2	11		
Hollie & Clinician Z	23.2	Model of care	Composite	44.59	2	2	0	1	2	0	1	0	0	0	0	0	8	7	**15**
					2	1	0	1	2	0	0	0	0	0	0	0	6		
Xue & Clinician T	24.8	Perineal trauma interventions	Composite	24.34	2	2	0	2	0	0	0	0	0	0	0	0	6	6	**12**
					2	1	0	1	1	0	0	0	1	0	0	0	6		
April & Clinician T	36.8	Induction of labour	Composite	30.01	1	1	0	1	1	0	0	0	0	0	1	1	6	5.5	**11**
					2	1	0	1	0	0	0	0	0	0	1	0	5		
Ria & Clinician Z	18.8	Spontaneous labour before elective caesarean	Composite	14.02	0	0	0	0	0	0	0	0	0	0	0	0	0	0	**0**
					0	0	0	0	0	0	0	0	0	0	0	0	0		
Libby & Clinician X	92.8	GBS swab	Composite	18.35	1	1	0	0	0	0	0	0	0	0	0	0	2	2	**4**
					1	0	0	0	1	0	0	0	0	0	0	0	2		
Maria & Clinician S	24.7	Iron supplementation	Composite	14.37	1	1	0	1	1	0	0	0	0	0	0	0	4	4	**8**
					1	1	0	1	1	0	0	0	0	0	0	0	4		
Wei & Clinician R	24.9	Timing of birth	Composite	22.14	1	1	0	1	1	0	0	0	0	0	0	1	5	4.5	**9**
					1	1	0	1	0	0	0	0	0	0	0	1	4		
Samantha & Clinician Q	22.6	Iron infusion	Composite	9.1	1	0	0	0	0	0	0	0	0	0	0	0	1	1	**2**
					1	0	0	0	0	0	0	0	0	0	0	0	1		
Alexandra & Clinician P	43	Vaginal birth after caesarean (VBAC)	Composite	16.55	2	1	0	1	0	0	0	0	0	0	0	1	5	6	**13**
					2	2	1	1	0	0	0	0	0	0	0	1	7		
Shania & Clinician P	61.2	Induction of labour	Review	8.03	1	0	0	0	0	0	0	0	0	0	0	0	1	1	**2**
					1	0	0	0	0	0	0	0	0	0	0	0	1		
Sana & Clinician P	35.7	Mode of birth	Review	10.48	2	2	0	2	2	0	0	0	0	0	0	0	8	7.5	**16**
					1	1	0	1	1	1	0	1	0	0	0	1	7		
Aysha & Clinician O	24	Participation in clinical trial	Composite	23.18	1	1	0	1	1	0	0	0	0	0	0	0	4	3	**6**
					1	0	0	0	1	0	0	0	0	0	0	0	2		
Josie & Clinician U	21.9	Treatment of genital herpes	Composite	16.22	2	2	0	1	1	1	1	0	0	0	0	0	8	8.5	**18**
					2	2	0	1	1	1	1	0	0	0	0	1	9		
Hazel & Clinician R	46.9	Iron studies pathology test	Review	21.01	1	0	0	1	1	0	0	0	0	0	0	0	3	3	**6**
					1	0	0	1	0	0	0	0	0	0	0	1	3		
Zoya & Clinician S	50.6	Glucose tolerance test	Composite	25.21	2	0	0	0	1	0	0	0	0	0	1	0	4	4	**8**
					2	0	0	0	1	0	0	0	0	0	1	0	4		
Halima & Clinician Q	24	Mode of birth	Composite	38.48	1	1	0	1	0	1	1	0	0	0	0	0	5	5.5	**11**
					1	1	0	1	1	1	1	0	0	0	0	0	6		
Iqra & Clinician O	56.5	Blood sugar monitoring	Composite	14.08	1	1	0	1	0	0	1	0	0	0	1	1	6	5	**10**
					1	1	0	1	0	0	0	0	0	0	0	1	4		
Leonie & Clinician O	40.2	Weight‐related investigations	Composite	9.05	1	0	0	0	0	1	0	0	0	0	1	0	3	3	**6**
					1	0	0	0	0	1	0	0	0	0	1	0	3		
Mya & Clinician O	42.3	Mode of birth	New	13.45	2	2	0	2	1	0	0	0	0	0	1	2	10	9.5	**20**
					2	2	0	1	1	0	0	0	0	0	1	2	9		
Item score sum					74	47	1	39	32	8	8	1	2	0	18	18			
Item score %**					75	45	0.9	37.5	30	8	8	0.9	1.9	0	17	17			

* OPTION12 score is calculated by determining the mean of the two raters' scores and transforming the mean score to a scale of 0–100 by dividing the mean score by 48 (the maximum OPTION12 score) and multiplying it by 100.

** Percentage item score = sum of item score divided by maximum score (104) and multiplied by 100.

The highest scoring items on the OPTION12 scale were Item 1 ‘clinician draws attention to an identified problem as one that requires a decision‐making process’ (71%), Item 2 ‘clinician states there is more than one way to deal with the identified problem’ (45%) and Item 4 ‘clinician lists options, which can include the choice of no action’ (37%). The lowest scoring items were Item 3 ‘clinician assesses the patient's preferred approach to receiving information to assist decision‐making’ (0.9%), Item 6 ‘clinician explores the patient's expectations about how the problem(s) are to be managed’ (0.9%) and Item 10 ‘clinician elicits the patients’ preferred level of involvement in decision‐making’ (0%).

Eighteen different index problems were discussed in the recorded appointments ranging from recommendations for diagnostic testing to planning mode of birth. The highest scoring index problems were decision making about mode of birth (OPTION12 score of 20) and treatment of genital herpes (OPTION12 score of 18). Induction of labour was both one of the highest and lowest scoring index problems, with one OPTION12 score of 24 and two OPTION12 scores of 2. Other low‐scoring index problems involved decision making about spontaneous labour before caesarean (OPTION12 score of 0), iron infusion (OPTION12 score of 2) and pathology testing for iron (OPTION12 score of 4 and 6).

### 3.3 OPTION12 Score and Body Mass Index

**Figure 2:**
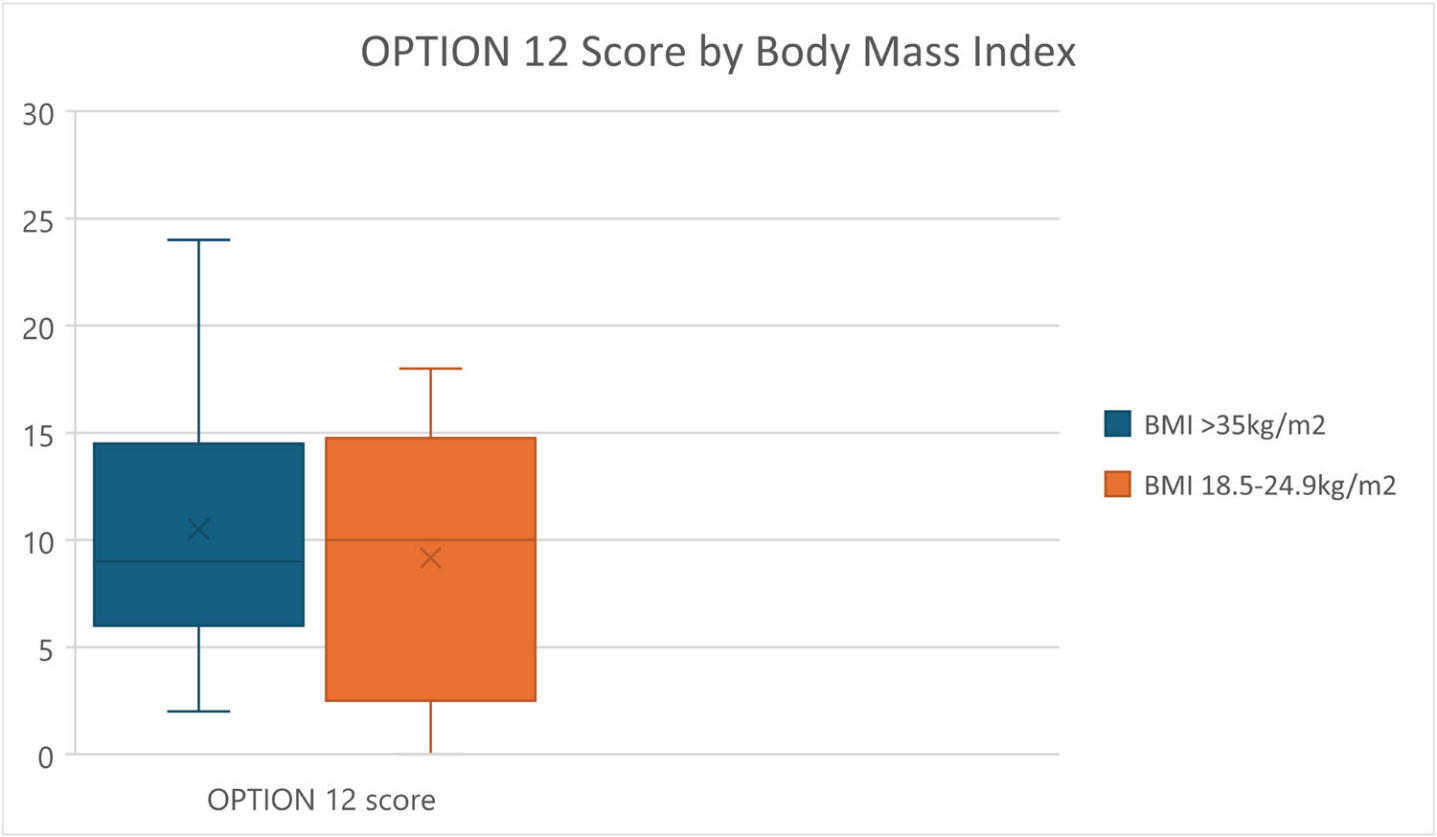
Box plot comparison of OPTION12 scores for women with high BMI and normal BMI.

**Figure 3:**
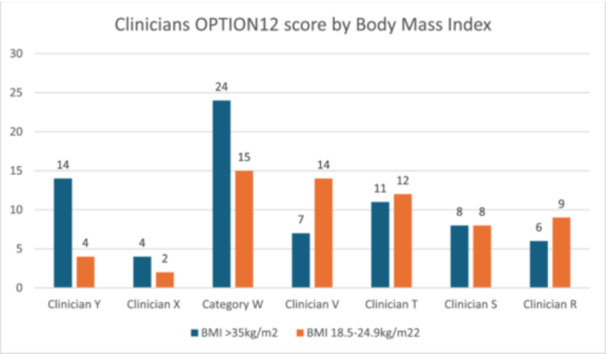
Comparison of clinicians OPTION12 scores with women with high BMI and normal BMI.

In the 26 recorded consultations, BMI did not appear to make a difference to the experience of shared decision‐making. Both the consultations with women with BMI ≥ 35 kg/m^2^ and the comparison group of women with BMI 18.5–24.9 kg/m^2^. demonstrated low OPTION12 scores of < 35. For the 12 women with BMI 18.5–24.9 kg/m^2^, the mean OPTION12 score was 9.2 and median 10.4. For the 14 women with BMI ≥ 35 kg/m^2^, the mean was 10.5 and median 9.3 (see Figure 2). Consultation duration was an average of 22 min for women with BMI 18.5–24.9 kg/m^2^ and 18 min for women with BMI ≥ 35 kg/m^2^. The OPTION12 scores for clinicians who saw women from both BMI groups can be seen in Figure 3.

### 3.4 Narrative Case Studies

Descriptive case studies provide insight into how shared decision‐making behaviour was, or was not, implemented in recorded consultations. Both the first and second case study involves decision making about induction of labour and includes Consultant Obstetrician Gynaecologists and multiparous women with BMI ≥ 35 kg/m^2^ in their third trimester of pregnancy. These case studies outline the highest and lowest scoring interactions with the index problem of induction of labour. In this study, the index problem of induction of labour had the greatest disparity between clinicians, with OPTION12 scores ranging between 2 and 24.

The first case study is the recorded interaction between Clinician W, a Consultant Obstetrician Gynaecologist, and pregnant woman Elise. This interaction scored 24 on the OPTION12 scale and the consultation lasted 31 min. In the recorded interaction, Clinician W was the first to draw attention to the index problem by stating: “*I would be very strongly considering the opportunity to look at an induction at around 39 to 40 weeks*” (Clinician W). Elise had stated earlier in the consultation that she wanted to avoid the prospect of caesarean section. In discussing the option of induction of labour, Clinician W reflects on this preference stating:

“Having heard you say that avoiding a caesarean is actually one of the things that you have prioritised in what you want at the end of your pregnancy, I think that maybe the possibility of exploring induction before we get to a point where the placenta's starting to run out of gas is going to be the best option for you.”

(Clinician W)

After incorporating Elise's stated preference into the proposed plan for induction of labour, Clinician W presented Elise with an opportunity to share her thoughts regarding the plan: “*Look, I'm not opposed to induction. Obviously, I'd like to see if my body does it naturally, but I said to my husband if they put induction back on the table I'm not going to try and be a hero…*” (Elise). Throughout the discussion, Clinician W asks for Elise's involvement, asking prompting questions like “*Is that reasonable?”* and responds with statements such as “*having heard you*”. Although Clinician W scored 0 for OPTION12 items 3, 9 and 10, a minimal attempt at shared decision‐making behaviour was observed for almost every other item on the OPTION12 scale.

In contrast, the second case study is the recorded interaction between Clinician P, a Consultant Obstetrician and Gynaecologist, and pregnant woman Shania. This interaction scored 2 on the OPTION12 scale and lasted 8 min. In the recorded interaction, Clinician P was the first to introduce the index problem stating, “*Once you've had the 36‐week scan, we'll probably aim for…somewhere around 38 weeks to book a date for you to come in so we can start labour”*. Clinician P then proceeded to ask Shania about her previous experience of induction of labour. Shania briefly discussed her last experience, focussing on the medical interventions. Clinician P ends the interaction the following way:

Clinician P: Of course. Probably, same thing will happen again. Likelihood is that it will be similar.

Shania: Yep.

Clinician P: Just so you're prepared.

Shania: Yep.

Clinician P: Okay, so, we'll see you in 2 weeks.

At no point in the interaction were alternative options discussed and at the conclusion of the appointment, the woman had not had the opportunity to share her expectations or concerns about the proposed decision for induction of labour. Both raters reviewing this interaction gave a score of zero for items 2–12, indicating that the only behaviour observed was the clinician drawing attention to an identified problem.

## 4 Discussion and Conclusion

### 4.1 Discussion

The aim of this study was to explore the experience of shared decision making for pregnant women and to understand whether BMI ≥ 35 kg/m^2^ limits access to shared decision making in antenatal care. There were two major findings. First, all women involved in the study experienced a low level of shared decision‐making behaviour. Second, BMI did not appear to influence the approach clinicians used for shared decision making.

This study demonstrated low levels of shared decision‐making behaviour by maternity clinicians during antenatal appointments. This study aligns with many other studies assessing use of shared decision making in health care, with very few available studies demonstrating exceptional shared decision‐making behaviour with high OPTION12 scores [50, 56, 57]. In a systematic review of 33 studies where OPTION12 was used, the majority of studies reported an average total OPTION12 score of < 35, demonstrating low implementation of shared decision making [50]. Although the OPTION12 scale is widely employed as a measure of shared decision making in research, this is only the second published study to use OPTION12 to assess implementation of shared decision making by maternity care providers. The first study, conducted in Italy in 2019 by Fersini et al. [39], explored shared decision‐making behaviour by obstetricians discussing mode of birth. The study of outpatient consultations identified a low level of shared decision‐making behaviour in appointments, aligning with the findings of this Australian study.

There are a number of possible explanations for the low OPTION12 scores by participants in this Australian study. A 2024 scoping review exploring shared decision making in an antenatal clinic identified barriers to shared decision making in practice [4]. First was the lack of continuity of care [4], and it is important to note that few of the participants in this study were seeing a clinician they had seen before, and none of the participants were involved in a continuity model of care like midwifery group practice or private obstetric care. Another barrier to shared decision making is limited time [4]. Consultations in this study varied in length from 8 to 44 min, however this included time spent undertaking physical assessment and discussing other topics. Like other studies in settings where multiple problems are raised within a consultation, it appears that shared decision making may be more difficult to implement in situations where several topics require discussion [50, 58].

In this study, BMI did not influence use of shared decision making in antenatal care. The case studies of women with BMI ≥ 35 kg/m^2^ demonstrate how shared decision‐making behaviour can be incorporated, or overlooked, in clinical decision making. Induction of labour is an obstetric intervention well suited to the shared decision‐making process, as there is often sufficient time to discuss options and alternatives [59, 60]. Induction of labour processes and methods vary, and there are many aspects of induction of labour that can be negotiated [60]. By introducing the plan for induction of labour as a statement rather than a question, both clinicians in the case studies demonstrate an authoritative approach to the recommendation of induction of labour. However, only one of the clinicians proceeded to involve the woman in the discussion and hear her expectations and concerns. The interaction between Clinician W and Elise which scored 24 on the OPTION12 scale lasted 31 min and the interaction between Clinician P and Shania scored 2 on the OPTION12 scale and lasted 8 min. While advocates for shared decision making state that it does not necessarily take more time from clinicians [46], time is frequently referenced as a barrier to shared decision making by clinicians and women [35, 45, 47]. Further quantitative research is required to understand the importance of time in antenatal consultations, including how much time is recommended to discuss complex index problems like induction of labour or timing of birth, compared to index problems that may take less time such as pathology tests or iron supplementation.

### 4.2 Strengths and Limitations

This study was an exploratory study with a limited sample size, meaning that it is not possible to draw generalisations from the data. Additionally, the range of index problems discussed in the recorded consultations make it difficult to compare the experience of shared decision making as interactions varied in content. Use of the OPTION12 scale could be considered a limitation as it does not measure consumer involvement or their perceived levels of involvement or satisfaction. Furthermore, as this is the first study to use OPTION12 to measure shared decision making in antenatal care in Australia and the first to explore the experience of shared decision making in antenatal clinic for women with BMI ≥ 35 kg/m^2^, more research is needed to build upon the limited findings of this study. Strengths of the study involve the exploration of a topic with very little existing evidence and the broad socio‐cultural demographic of women represented, with varied gestations and BMI.

### 4.3 Conclusion

This exploratory study illustrates that shared decision‐making behaviour is limited in the antenatal clinic setting in Australia for all women, regardless of BMI. The low levels of shared decision‐making behaviour demonstrated in this study suggest implementation of shared decision making which is not aligning with the mandated requirement for shared decision making instituted by the Australian Commission on Safety and Quality in Health Care. It is evident that more work needs to be done to ensure shared decision making becomes embedded in clinical practice to improve the involvement of all pregnant women seeking hospital‐based antenatal care.

## Author Contributions

**Madeline Hawke:** conceptualisation, investigation, funding acquisition, writing–original draft, methodology, writing–review and editing, formal analysis, data curation, project administration, software. **Linda Sweet:** methodology, supervision, writing–review and editing, resources, validation. **Julie Considine:** supervision, writing–review and editing, funding acquisition, methodology, resources, validation.

## Ethics Statement

This study has received ethics approval from the Royal Melbourne Human Research Ethics Committee (HREC/94723/MH‐2023). The date of approval was 24 May 2023. The project title for ethics approval was: Maternity clinicians communication with pregnant women and birthing people in antenatal clinic.

## Conflicts of Interest

The authors declare no conflicts of interest.

## Data Availability

The data that support the findings of this study are available on request from the corresponding author, MH. The data are not publicly available due to their containing information that could compromise participant privacy and confidentiality.
